# The London Hospital

**Published:** 1902-09-13

**Authors:** 


### THE LONDON HOSPITAL.

PROGRESS OF THE BUILDING OPERATIONS—APPOINTMENT OF TWO ASSISTANT PHYSICIANS.

A QUARTERLY general and special Court of the London Hospital was held at the hospital on Wednesday, 3rd inst., to receive the report of the House Committee and to appoint two assistant physicians. Mr. J. H. Hale was voted to the chair. The House Committee reported that during the last three months continued activity and substantial progress have been shown in all the building operations at the hospital. The new "Cambridge" and "Gloucester" wards have been opened for some weeks and give universal satisfaction. An experiment has been made in tiling the walls with opalite; and the Committee estimate that the saving in cleaning will pay 3 per cent. on the cost of the tiling. The new out-patient department is being rapidly completed, and the waiting hall was so far advanced on July 5th that the committee was enabled to offer it to the local committee of the King's Dinner Fund, and no less than 1,100 people were accommodated in the hall and adjoining rooms. The receiving room is finished and ample examining rooms are now provided for any emergency. The Hermann de Stern Convalescent Home at Felixtowe has been opened and kept as full as its endowed funds will permit. Not more than 24

patients can be taken at one time, although there is room for 28 or 29 beds. The care and attention bestowed on the patients under the able management of Miss Wamsley are spoken of most highly. The tunnel to the new out-patient department is now almost completed, and numerous alterations are in full swing in many of the other wards and in the west wing and the basement. The committee has also accepted a tender for £23,870 for the new laundry. Before the next quarterly meeting the committee hopes to be installed in the new board room which is taking the place of the old "Crossman" ward. An alteration has been made in the visiting days, which are now Wednesdays and Sundays and for Hebrews Saturdays. The hospital has been visited recently by the Maharajah of Jaipur and Sir Jamsetjee Jejeebhoy (the latter a grandson of him after whom one of the wards has been named), and both expressed satisfaction with all they saw. A most satisfactory report has been received from Dr. Hayward with regard to the examination of the nurses, and great praise is due to the sisters for their able tuition and commendable results. The committee record their pride in the fact that Sir Frederick Treves, who performed the operation on the King, and Dr. Frederick W. Hewitt, who gave the anæsthetic, are both on the staff of the London Hospital; the institution was also called upon to provide a nurse, and the committee are proud to learn that Nurse Haines acquitted herself exceedingly well. The resignation of Mr. Mark Hovell was received with sincere regret, and the committee record their high appreciation of the valuable work done by him for so many years in the hospital, and proposed that he be elected a consulting aural surgeon. The death has occurred in the hospital of Mr. A. W. Fisher, a most promising student, who filled the post of a clinical assistant in the out-patient department and gave promise of a brilliant future in his profession. With a view to checking the large and increasing expenditure in the dispensary department new rules have been drawn up which it is hoped will have the desired effect. After alluding to a smart piece of work by the hospital fire brigade in extinguishing an outbreak of fire, the committee go on to say that they have received from the Sunday Fund the largest amount yet apportioned to any hospital, viz, £6,181 5s. For the compulsory sale of a site in Myrdle Street, Mile End, to the London School Board, the hospital was awarded £27,000 as compensation; but the committee would rather have the site. In spite of some generous legacies the hospital is still indebted for a loan of £20,000 to their bankers. There being a vacancy on the House Committee, Baron Herbert de Stern was proposed to fill the position. Among the donations were £1,000 from the Worshipful Company of Grocers, £250 chapel offertory, and £100 each from Mr. Arthur Cotes and D. C. Stiebel. The following legacies have also been received:—W. Brocksopp, £300; A. Titmuss, £450; Mrs. Bates, £494; Matthew Whiting, £2,500; Thos. Whitfield, £3,000; H. M. Turnor, £4,500; David Cohen, £200; and Alfred Bilton, £100.

The House-Governor (Mr. G. Q. Roberts) reported that during the quarter 539 patients had been discharged cured, 2,836 discharged relieved, and 318 had died, making a total of 3,243 patients in the period, during which 20,189 out-patients had also received attention. The number of patients in hospital that day was 700, the daily average of the three months being 668, while the daily average for the nine months worked out at 676.

The Chairman, in moving the adoption of the report, drew attention to the number of patients in hospital, and said the governors would realise the great anxiety it was to the committee to secure funds to meet the necessary expenditure of such a large institution as theirs. That day they had the announcement of a magnificent gift to the King's Hospital Fund, and he was sure all interested in the hospital would be delighted with the news, as the London Hospital, in common with other such institutions, would benefit by the gift. As was mentioned in the report, they were forced to sell their Myrdle Street site to the London School Board, and for the handsome sum obtained they had to thank their counsel, who eloquently pleaded their cause at the inquiry. They were all satisfied with the amount awarded, though they would have preferred to retain the site.

Sir Edmund Currie seconded the motion, which was carried.

The special business of the Court was the appointment of two assistant physicians. Dr. A. L. Smith was nominated as assistant physician and Dr. Sequeira was proposed for a similar position in the skin department. In support of the house committee's recommendation of these gentlemen for the posts, the chairman spoke of their brilliant careers, and in the case of Dr. Sequeira referred to the excellent work he had done in connection with the light treatment of lupus, the lamps for which, it may be recalled, were presented by her Majesty the Queen. Both gentlemen were unanimously appointed and briefly returned thanks for the honour conferred upon them.

This concluded the business, and the chairman was cordially thanked for his services.

